# Effects of human probiotics on memory and psychological and physical measures in community-dwelling older adults with normal and mildly impaired cognition: results of a bi-center, double-blind, randomized, and placebo-controlled clinical trial (CleverAge biota)

**DOI:** 10.3389/fnagi.2023.1163727

**Published:** 2023-07-07

**Authors:** Ales Bartos, Josefina Weinerova, Sofia Diondet

**Affiliations:** ^1^Department of Neurology, Third Faculty of Medicine, Charles University, Prague, Czechia; ^2^Department of Neurology, Faculty Hospital Královské Vinohrady, Prague, Czechia; ^3^National Institute of Mental Health, Klecany, Czechia; ^4^First Faculty of Medicine, Charles University, Prague, Czechia

**Keywords:** probiotics, clinicaltrials.gov NCT05051501, memory, depression, gut microbiota, cognitive impairment, Alzheimer disease

## Abstract

**Objectives:**

This study presents results of our randomized clinical trial studying the effect of human probiotics on memory and psychological and physical measures following our study protocol registered at clinicaltrials.gov NCT05051501 and described in detail in our previous paper.

**Methods:**

Community dwelling participants aged between 55 and 80  years were randomly assigned to receive a single dose of 10^6^ colony-forming units of human *Streptococcus thermophilus* GH, *Streptococcus salivarius* GH NEXARS, Lactobacilus plantarum GH, and *Pediococcus pentosaceus* GH or placebo. A cross-over design allowed each group to receive probiotics and placebo for 3  months each in reverse order. A small subset of participants was examined online due to the COVID-19 pandemic. After 6  months a small number of volunteers were additionally assessed after 2  months without any intervention. Primary outcome measures included changes in cognitive functions assessed using brief tests and a neuropsychological battery and changes in mood assessed using validated questionnaires. Secondary outcome measures included changes in self-report and subjective measures using depression and anxiety questionnaires, seven visual analog scales of subjective feelings (memory, digestion, etc.), and physical performance.

**Results:**

At baseline, the probiotic-placebo group A (*n* = 40, age 69 ± 7 years, education 16 ± 3 years, 63% females, body mass index 28.5 ± 6, subjective memory complaint in 43%) did not differ from the placebo-probiotic group B (*n* = 32) in any of the sociodemographic characteristics and evaluated measures including cognitive status. At follow-up visits after 3, 6, and 8  months, no cross-sectional differences in any of the measures were found between the groups except worse sentence recall of the ALBA test after 3  months of probiotic use. Score changes were not observed for all cognitive tests but one in any group between visits 1 and 3 and between visits 3 and 6. The only change was observed for the TMT B test after the first three months but no change was observed after the second three months.

**Conclusion:**

The treatment with human probiotics and prebiotics did not improve cognitive, affective, or physical measures in community-dwelling individuals with normal or mildly impaired cognitive functions.

**Clinical trial registration:**

clinicaltrials.gov, identifier NCT05051501.

## Introduction

Is there a link between gut microbiota and brain function? The answer to this question has been extensively studied in recent years (Trejo-Castro et al., 2022). If this pathway exists, then manipulating gut microbiota may affect brain functions. Gut microbiota can be influenced by polymedication, lifestyle, or interventions that include probiotic intake, diet, and fecal transplantation (Białecka-Dębek et al., 2021; Sánchez-de-Lara-Sánchez and Sánchez-Pérez, 2022).

Forgetfulness and memory impairment increase with aging. Depression is also present among the elderly. Therefore, it would be beneficial to maintain or even improve cognitive and affective functions with probiotics. This natural treatment was used in elderly participants with various levels of cognitive functions. While some meta-analyses found partial favorable effects of probiotic supplementation (Lv et al., 2021; Sánchez-de-Lara-Sánchez and Sánchez-Pérez, 2022; Xiang et al., 2022), others did not (Krüger et al., 2021; Tahmasbi et al., 2022). In general, patients with dementia do not benefit (Krüger et al., 2021; Tahmasbi et al., 2022) and patients with mild cognitive impairment (MCI) show statistically significant improvement only in some specific tests and sub-analyses (Lv et al., 2021; Sánchez-de-Lara-Sánchez and Sánchez-Pérez, 2022). Results in healthy older adults were negative with a few exceptions (Ohsawa et al., 2018; Tahmasbi et al., 2022). Some studies also reported the absence of mood changes following probiotic treatment (Benton et al., 2007; Chung et al., 2014; Inoue et al., 2018; Ohsawa et al., 2018; Louzada and Ribeiro, 2020). This is in contrast to a systematic review suggesting that treatment with probiotics may improve symptoms associated with major depressive disorder (Wallace and Milev, 2017).

Due to the controversies within this evolving area of study, we decided to design a randomized clinical trial with a cross-over design (Bartos et al., 2022). Studies focusing on cognitively normal or mildly affected older adults are scarce (Tahmasbi et al., 2022). We assumed that probiotic effect could be observed particularly in individuals with mildly impaired cognitive functions and serve as a form of prevention against further deterioration (Sánchez-de-Lara-Sánchez and Sánchez-Pérez, 2022). The aim of our study was to evaluate the effect of probiotics with prebiotics on memory and psychological and physical measures in community-dwelling individuals. We hypothesized that participants would benefit from human probiotics and prebiotics taken for 3 months.

## Participants and methods

The detailed protocol for this study can be found in our previous paper (Bartos et al., 2022). Here we briefly describe participants´ characteristics and procedures. Our study was carried out at two centers, namely the University Hospital Kralovske Vinohrady (UHKV), Charles University, Third Faculty of Medicine, Prague, and the National Institute of Mental Health (NIMH) in Klecany near Prague, Czech Republic from January 2021 to April 2022.

### Ethics statement

This trial was performed in accordance with the Declaration of Helsinki. All participants signed their informed consent before beginning the study. The study was approved by the Ethics Committees of NIMH Klecany (No 78 and 165/20) and UHKV Prague in 2020 (No EK-VP 17/0 and 1/2020) and was registered on the clinicaltrials.gov portal with registration number NCT05051501.

### Inclusion and exclusion criteria of eligibility for the study

The participants were aged between 55–80 years, had Czech as a native language, had preserved activities of daily living, and had good sight and hearing. Participants were excluded from the study if they had diseases or conditions of the digestive tract, neurological brain diseases, psychiatric diseases or treatment, oncological diseases, or had used cognitive enhancers or other probiotic supplements within 3 months prior to the study onset. Depression was not an exclusion criterion.

### Participants

**Figure 1:**
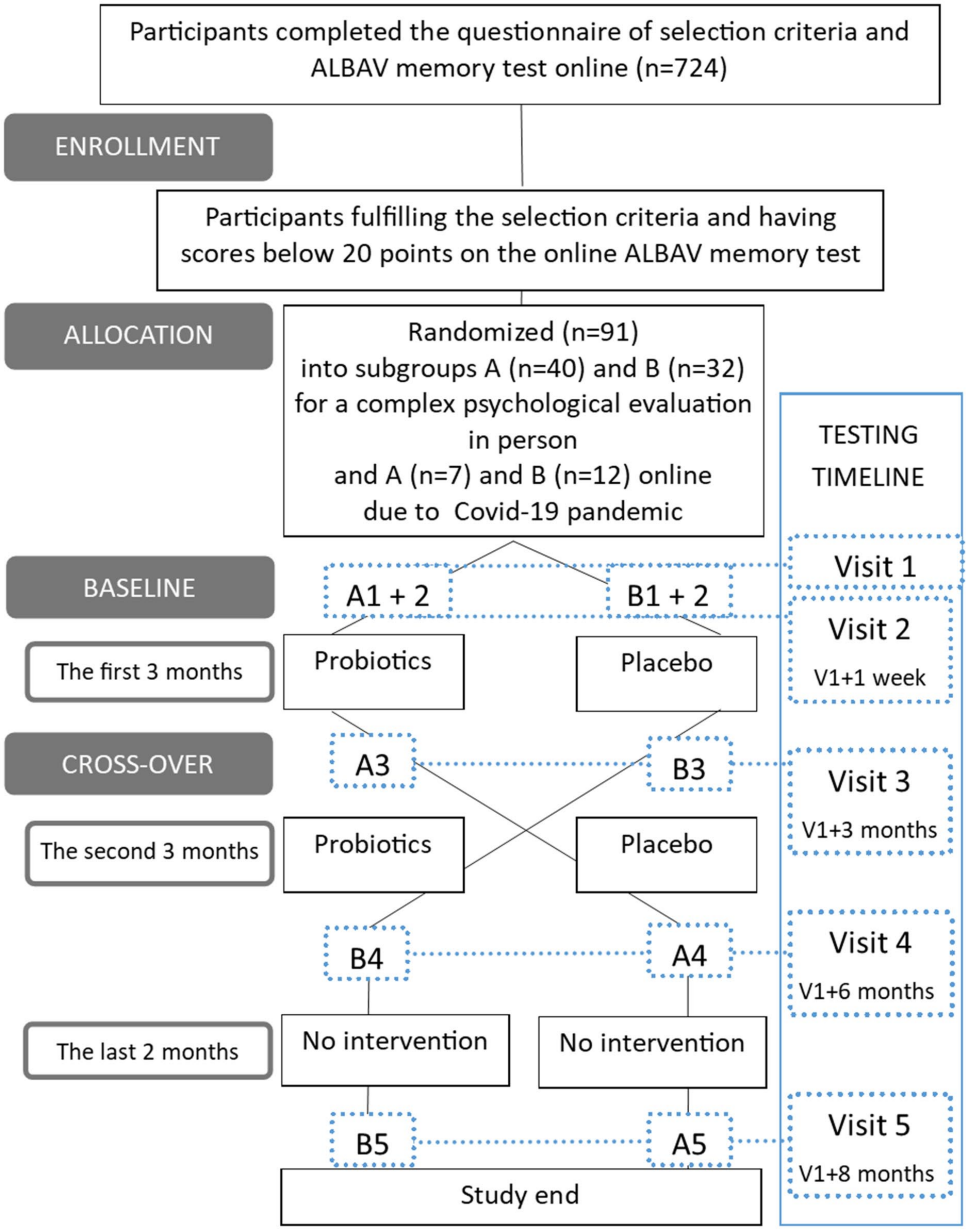
Flowchart of participant recruitment and study design. The timeline shows all the visits connected by a dotted line to identify data subsets by subgroups and time of testing. A—a group starting with a probiotic period followed by a placebo one; B—a group starting with a placebo period followed by a probiotic one; Visit 1-5 – visits with psychological and other evaluations.

Participants were recruited using an online form detailing all inclusion and exclusion criteria and a short online memory test named ALBAV to increase chances for identifying those with memory impairment prior to the trial onset (Bartos and Krejcova, 2022; Bartos and Krejcova, 2023). The process of recruitment, randomization, and examinations is shown in Figure 1.

The final number of participants (*n* = 91) was randomly divided into two subgroups (group *pro*biotics first, *pla*cebo later PROPLA (A): *n* = 47, group *pla*cebo first, *pro*biotics later PLAPRO (B): *n* = 44). A small subgroup of participants refused to come to in-person assessments due to fear of COVID-19 during the pandemic in the Czech Republic (Dvořáková et al., 2022). Therefore, they participated online (*n* = 19; online group A PROPLA: *n* = 7, online group B PLAPRO: *n* = 12). They were assessed with cognitive tests remotely via computers using monitors, web cameras, speakers, and microphones. We described our experiences with this type of distant testing previously (Polanská and Bartoš, 2022). The rest of the procedures were performed in person and included completing self-report questionnaires, tests of physical performance, and biological sample collection. All procedures were performed in person for the remaining 72 individuals.

Given this sample size, we were able to detect an effect size of Cohen’s d = 0.43 in within-subject analyses and Cohen’s d = 0.59 in cross-sectional comparisons (assuming α = 0.05 and power = 0.8). Details of the power calculation are presented in our study protocol article (Bartos et al., 2022).

### Study design

Our study was a randomized, double-blind, placebo-controlled trial with a cross-over design. The study design is graphically shown in Figure 1.

Each participant was examined at four visits to the University Hospital Kralovske Vinohrady, Prague, or at the National Institute of Mental Health, Klecany, Czech Republic.

A probiotic intervention was assessed with a comprehensive evaluation including the following aspects: brief cognitive tests, neuropsychological battery, self-report questionnaires, visual scales, physical performance, actigraphy, and blood, urine, and stool samples. Biological material was subject to measurements of neurofilament light concentrations in serum and metabolomic High-Performance Liquid Chromatography Mass Spectrometry (Bridel et al., 2019; Bartos et al., 2022; Fialová et al., 2022). The results from these analyses will be published in future separate papers. Here we present the psychological and physical results.

Both groups of participants received placebo and probiotics for 3 months each but differed in the order of application. There was no wash-out period between probiotic and placebo use. Both participants and administrators were blinded to the type and the order of the intervention. All of the measurements were taken at up to five visits. The initial procedures were performed on two visits (V1 + V2) due to logistic reasons.

Only a subset of the participants continued without any intervention for a further 2 months after the trial termination to find out the long-term effects of microbiome changes in an additional follow-up visit (*n* = 16 in group A, *n* = 15 in group B). The interval was slightly shorter than originally planned in the protocol paper (Bartos et al., 2022) because the COVID-19 pandemic interfered with our grant and the grant ended. All participants at each visit were asked about the adverse effects of the placebo/treatment using a structured list of relevant symptoms.

Participants received compensation of 3,000 CZK for their study participation at the end of the study (that is the equivalent of 123 EUR or 140 USD at current currency exchange rate).

### Probiotic intervention

Each participant received a brochure with detailed information and instructions regarding the project, examinations at institutions, specimen sampling at home, and correct intake of tablets. They were asked a number of open-ended questions about several measures at visits 3 and 4.

We randomly assigned participants to receive a single dose of 10^6^ colony-forming units of human *Streptococcus thermophilus* GH, *Streptococcus salivarius* GH NEXARS, Lactobacilus plantarum GH, and *Pediococcus pentosaceus* GH or placebo in one probiotic/placebo tablet along with two fiber/placebo fiber tablets once a day. The time of consumption was up to the participants. Unlike other products available on the market, which are usually of bovine origin, our probiotics were manufactured using human-stemmed lines.

The placebo for probiotics was composed of semi-coarse wheat flour, starch, maltodextrin, and magnesium stearate. The fiber placebo was composed of cellulose, maltodextrin, and stearate magnesium. The placebo tablets were identical visually and taste-wise to the probiotic tablets. Probiotic supplements, prebiotics, and placebo were provided by NEXARS (Brno, Czech Republic).

### Primary outcome measures

We assessed the cognitive functions of participants using a combination of brief tests and a neuropsychological battery. Tests and questionnaires were administered in the same order at all four testing sessions by the same administrator if possible, to decrease assessment variability. A detailed description of all measures can be found in our protocol paper (Bartos et al., 2022).

#### Brief cognitive tests

The brief tests included the Clock Drawing Test (CDT), our newly developed Amnesia Light and Brief Assessment (abbreviated from the initial letters as ALBA), and our in-house Assessment Battery of Cognition (abbreviated from the initial letters as ABACO) which consists of five brief tests: (1) the Reading Encrypted Sentences subtest, (2) sentence learning and recall, (3) verbal fluency test, (4) the PICture Naming and Immediate Recall (abbreviated from the initial letters as PICNIR) test, and (5) the Five or Four-line test (Bartos et al., 2022). The CDT was evaluated using our developed and validated scoring system called BaJa (Bartos et al., 2016).

The ALBA test consists of repeating a sentence of six words, performing and later recalling six gestures, and finally recall of the words of the original sentence (Bartos, 2019; Bartos and Diondet, 2020). Validation of the ALBA test was just introduced in English (Bartos and Diondet, 2023). The ALBA educational video is freely available at: https://www.youtube.com/watch?v=LyCuWc0-Gro.

In the PICNIR test, the first task is to write down the names of 20 black and white pictures and then to recall and write as many picture names as possible in one minute (Bartos, 2016; Bartos and Polanska, 2021). The PICNIR educational video is freely available at https://www.youtube.com/watch?v=cbJGtPG-nVA.

Two innovative ALBA and PICNIR tests are easy to perform and evaluate, but challenging for the evaluated person at the same time, they are very brief lasting up to five minutes (only 6–8 minutes together) and are used to detect mild cognitive deficits, especially short-term episodic or long-term semantic memory, aphasia, and dysgraphia. They may be sensitive enough to detect subtle and incipient impairment changes in offspring of patients with dementia (Bartos, 2021).

More information about all brief tests is in our study protocol paper (Bartos et al., 2022).

#### A comprehensive battery of neuropsychological tests

Two similar neuropsychological batteries were prepared for in-person and online assessments of the baseline and follow-ups. A more detailed description of all the tests administered in person and online and their order are reported in our previous study protocol paper (Bartos et al., 2022). The battery included the following validated measures: (1) three alternative versions of the Rey Auditory Verbal Learning Test (RAVLT) to evaluate verbal memory; (2) the same version of the Trail Making Test (TMT) consisting of part A to evaluate the processing speed, attention, and visuospatial ability and part B to assess mental flexibility as a part of executive functions; (3) the Digit Symbol subtest from The Wechsler Adult Intelligence Scale III (WAIS-III) to assess processing speed; and (4) the category (animal) fluency test in-person and online and phonemic fluency tests with initial letters NKP during online testing used to evaluate executive functioning, language, and semantic memory. In addition, online testing also included the Digit Span subtest from WAIS-III which measures the capacity of short-term verbal memory and working memory (Lezak et al., 2012; Preiss et al., 2012).

#### Self-report subjective questionnaires of depression and activities of daily living

Participants filled in the Geriatric Depression Scale (GDS) questionnaire and the Functional Activities Questionnaire (FAQ-CZ) to assess their mood and activities of daily living (Pfeffer et al., 1982; Yesavage et al., 1982).

### Secondary outcome measures

Physical body characteristics were evaluated using the Beurer BF 950 diagnostic scale which provided the following data: weight, Body Mass Index (BMI), body water percentage, muscle mass percentage, and bone weight.

#### Self-reported and subjective measures

Depression and anxiety were evaluated with the second version of the Beck Depression Inventory and the Beck Anxiety Inventory (BDI-II, BAI) (Beck et al., 1988, 1996). Additionally, participants were asked to express their subjective feelings regarding their memory, digestion, overall health, sleep, anxiety, tiredness, and pain using seven Visual Analog Scales (VAS) ranging from 1 to 10 points. Participants were also asked about possible side effects of probiotics in a questionnaire.

#### Physical performance measures

Participants completed three tests of physical performance (number of lifts of 1 kg dumbbell from extended arm to shoulder in 30 s, walking time of 2×17m distance, and number of stand ups from sitting position in a chair in 30 s).

### Statistical analyses

Data are presented as means with standard deviations or as percentages. Results of sociodemographic, test, and questionnaire scores and other measures were compared using the unpaired *t*-test for continuous variables between the two groups at baseline and follow-up visits. Score differences in the neuropsychological tests and two brief memory tests, ALBA and PICNIR, were calculated between results at visit 1 and 3 and between those at visit 3 and 4. These differences were compared between the two groups using the unpaired *t*-test. The Chi-squared test was used to compare categorical data at baseline and to evaluate adjacent symptoms monitored during the clinical trial, mainly gastrointestinal. Multiple regression analysis was performed to assess the influence of age and gender on differences of RAVLT and TMT B between visit 1 and 3 and visit 3 and 4 in each group PROPLA and PLAPRO. All the statistical analyses were performed using Statistica software. A level of *p* < 0.05 was considered statistically significant.

## Results

The probiotic-placebo group A and the placebo-probiotic group B matched in baseline sociodemographic characteristics, cognitive categorization, and apolipoprotein E status as shown in Table 1. At baseline, neither group differed in scores in brief cognitive tests and neuropsychological tests, self-report and subjective questionnaires or scales, personal body characteristics, and physical fitness measures. Thus, the two groups were well matched at baseline visit 1. Online subgroups were also well-matched for sociodemographic variables (Supplementary Table S1).

**Table 1:** Participant characteristics and comparisons of socio-demographically matched groups at baseline.

	Group PROPLA (A)	Group PLAPRO (B)	Value of *p*
Number of participants	40	32	
Age (years)	69.5 (65.5–75) 69 ± 7	72.5 (69–76) 71.5 ± 6	n.s.
Education category	2 (8%)/3 (43%)/4 (50%)	1 (3%)/3 (41%)/4 (56%)	n.s.
Education (years of schooling)	17 (13–18.5) 16 ± 3	16.5 (15–18) 17 ± 3	n.s.
Female participants (percent)	25 (63%)	18 (56%)	n.s.
Height (cm)	169 (161–178) 169 ± 9	170 (164–178) 170.5 ± 7.5	n.s.
Weight (kg)	72 (66–91) 79 ± 20	78.5 (65–89) 78 ± 14.5	n.s.
BMI	27.5 (25–30) 28.5 ± 6	26.5 (24.5–30) 27 ± 3.5	n.s.
Hearing ability (percent)	100%	100%	n.s.
Visual ability (percent)	100%	100%	n.s.
Evaluation site UHKV/NIMH (percent)	68%/33%	69%/31%	n.s.
Subjective memory complaints according to GDS question 10 (percent)	17 (43%)	19 (59%)	n.s.
Cognitive categorization (cognitively normal)/(mild cognitive impairment)	72%/28%	75%/25%	n.s.
Apolipoprotein E allele 1 (2/3) (percent)	5%/95%	13%/87%	n.s.
Apolipoprotein E allele 2 (3/4) (percent)	77%/23%	84%/16%	n.s.

Data are presented as median and interquartile range (25–75 percentile) and mean ± standard deviation. Group PROPLA (A): probiotics first, placebo later; group PROPLA (B): placebo first, probiotics later; Education (category): 1 = Primary school, 2 = High school without a General Certificate of Education (GCE), 3 = High school with GCE, 4 = University. BMI, body mass index; UHKV, University Hospital Kralovske Vinohrady; NIMH, National Institute of Mental Health; GDS, Geriatric depression scale; *p*, probability of a group result difference, n.s., not significant.

### Efficacy assessments

#### Cross-sectional comparisons

At follow-up visits after 3, 6, and 8 months, no cross-sectional differences in any measures were found between the two groups with one exception. The number of correctly recalled words of a sentence after distraction in ALBA was significantly lower in the group taking probiotics for 3 months compared to those on placebo, i.e., worsening due to probiotics (*p* = 0.01). However, a similar difference was not observed in the other group after the following 3 months of reverse intake. Baseline and follow-up results of both groups and their comparisons are shown for brief cognitive tests in Table 2, for neuropsychological tests in Table 3, for questionnaires and visual analog scales in Table 4, and for personal body characteristics and physical performances in Table 5.

**Table 2:** Results of brief cognitive tests and their comparisons between the groups PROPLA and PLAPRO at several visits.

Visit	Visit 1 (plus Visit 2) baseline	Visit 3 (assessments after three months)	Visit 4 (assessments after three months)	Visit 5 (assessments after two months)
	Group PROPLA (A1)/group PLAPRO (B1)	Group PROPLA after probiotics (A3)/group PLAPRO after placebo (B3)	Group PROPLA after placebo (A4)/group PLAPRO after probiotics (B4)	Group PROPLA without intervention (A5)/group PLAPRO without intervention (B5)
Number of participants	40/32	37/30	37/30	16/15
Intervals from baseline assessments (days)	0	98 ± 10/96 ± 10	98 ± 20/96 ± 15	56 ± 11/57 ± 14
CDT evaluated by BaJa scoring (0–5 points)	4.5 ± 1/4.5 ± 1	4.5 ± 1/4.5 ± 1	4.5 ± 1/5 ± 0.5	4.5 ± 1/4.5 ± 1
ALBA test version	version A	version B	version A	version B
ALBA Sentence encoding-number of correctly repeated words of the sentence (0–6 words)	6 ± 0.5/6 ± 0.5	6 ± 0.5/6 ± 1	6 ± 0.5/6 ± 0.5	6 ± 0.5/6 ± 0.5
ALBA Sentence recall – number of correctly recalled words of the sentence after distraction using the TEGEST (0–6 words)	4.5 ± 2/4.5 ± 2	3 ± 2/4 ± 2* (*p* = 0.01) worsening due to probiotics	5 ± 1.5/4.5 ± 1.5	4 ± 2/4 ± 2
ALBA TEGEST – initial demonstration of six gestures according to instructions (0–6 gestures)	6 ± 0/6 ± 0	6 ± 0/6 ± 0	6 ± 0.5/6 ± 0	6 ± 0/6 ± 0
ALBA TEGEST gesture recall – number of correctly recalled gestures (0–6 gestures)	4.5 ± 1/4 ± 1	5 ± 1/4.5 ± 1	4.5 ± 1/4.5 ± 1	5 ± 1/4 ± 1.5
ALBA memory score– the sum of the number of correctly recalled sentence words and gestures (0–12 points)	9 ± 2/9 ± 2	7.5 ± 2.5/8 ± 2.5	9.5 ± 2/9 ± 2	8.5 ± 2/8 ± 3
ABACO test version	version 1	version 2	version 1	version 2
ABACO Reading Encrypted Sentences subtest (0–3 points)	2.5 ± 1/2.5 ± 1	2.5 ± 1/2 ± 0.5	2.5 ± 1/2.5 ± 0.5	2.5 ± 0.5/2 ± 1
ABACO sentence learning/encoding first trial (0–10 words)	7 ± 2/8 ± 2	7.5 ± 2/7 ± 2	8 ± 1.5/9 ± 1	8 ± 1.5/8 ± 2
ABACO sentence learning/encoding second trial (0–10 words)	9 ± 1/9 ± 1* (8.6 *vs* 9.2) (*p* = 0.02)	9 ± 1/9 ± 1	9 ± 1/9.5 ± 1* (9.0 *vs* 9.6) (*p* = 0.04)	9 ± 1/9 ± 1
ABACO sentence immediate recall (0–10 words)	7.5 ± 2/8 ± 2	7.5 ± 2/8 ± 2.5	8.5 ± 1.5/9 ± 1	8.5 ± 1/8 ± 2.5
ABACO sentence delayed recall (0–10 words)	6 ± 2/7 ± 2	5.5 ± 2/6 ± 3	6.5 ± 2.5/7 ± 3	7 ± 2/6 ± 3
ABACO verbal fluency task in 30 s	11 ± 3/13 ± 2 * (11.3 *vs* 12.7) (*p* = 0.03)	12 ± 2.5/12 ± 3	12.5 ± 3/12 ± 2.5	13.5 ± 3/13 ± 4
ABACO the PICNIR Picture naming mistakes – number of mistakes or unnamed pictures (0–20 pictures)	0.5 ± 1/0.5 ± 1	0.5 ± 0.5/0.5 ± 0.5	0.5 ± 1/0.5 ± 0.5	0.5 ± 0.5/0.5 ± 0.5
ABACO the PICNIR Picture naming recall – number of correctly recalled pictures (0–20 words)	8.5 ± 3/8 ± 3	9 ± 2/8.5 ± 2.5	9 ± 2/9 ± 3	10 ± 3/9 ± 3
ABACO the Four or the Five-line test score (0–4 points)	3 ± 1/2.5 ± 1	3.5 ± 1/3 ± 1	3 ± 1/3.5 ± 1	3.5 ± 0.5/3.5 ± 1
ABACO total score (0–35 points)	25 ± 6.5/26 ± 6	26 ± 6/26 ± 7	27.5 ± 6/27.5 ± 6	29.5 ± 4/26.5 ± 8

The differences of all the brief cognitive tests were not significant between the two groups at every visit except for those in bold and with asterisks. Data are presented as mean ± standard deviation. The values in the table are rounded to half (=0.5). Precise calculated values with one decimal place in the brackets are only in cases with significant differences. Group PROPLA (A): probiotics first, placebo later; group PROPLA (B): placebo first, probiotics later; CDT, the Clock Drawing Test; BaJa, scoring by Bartos and Janousek; ALBA, the Amnesia Light and Brief Assessment test; TEGEST, the test of gestures; RAVLT, the Rey Auditory Verbal Learning Test; TMT A and B, Trail Making Test parts A and B; ABACO, The Assessment BAttery of Cognition; PICNIR, the PICture Naming and Immediate Recall; *p*, probability.

**Table 3:** Results of neuropsychological tests and their comparisons between the groups PROPLA and PLAPRO at several visits.

Visit	Visit 1 (plus Visit 2) baseline	Visit 3 (assessments after three months)	Visit 4 (assessments after three months)	Visit 5 (assessments after two months)
	Group PROPLA (A1)/group PLAPRO (B1)	Group PROPLA after probiotics (A3)/group PLAPRO after placebo (B3)	Group PROPLA after placebo (A4)/group PLAPRO after probiotics (B4)	Group PROPLA without intervention (A5)/group PLAPRO without intervention (B5)
Number of participants	40/32	37/30	37/30	16/15
Intervals from baseline assessments (days)	0	98 ± 10/96 ± 10	98 ± 20/96 ± 15	56 ± 11/57 ± 14
Score of Category Fluency Test (animals in one minute)	24 ± 5.5/24 ± 5.5	25 ± 6/25 ± 6	26 ± 5/26 ± 6	25.5 ± 4/22.5 ± 6
RAVLT test version	version 1	version 2	version 3	version 1
Total number of words in sets A 1–5 in RAVLT (0–75 words)	46 ± 10/47 ± 8	48 ± 9/46.5 ± 10	51 ± 10/50 ± 8	56 ± 7/49.5 ± 12
Percentile in sets A 1–5 in RAVLT (0–100)	44 ± 28.5/52 ± 27	55 ± 28/51 ± 31	58 ± 28/64 ± 28	72 ± 24/59 ± 32
Number of words on delayed *recall* in RAVLT (0–15 words)	8.5 ± 3/8 ± 3	9.5 ± 3/9 ± 4	9.5 ± 3/9.5 ± 3.5	11 ± 3/9 ± 4.5
Percentile of delayed *recall* in RAVLT (0–100)	39 ± 24/44 ± 27	51.5 ± 28/47 ± 32	51.5 ± 31/57 ± 29	61 ± 30/55.5 ± 37
Duration of TMT A (seconds)	41 ± 15/43 ± 13	38 ± 12/41 ± 12	38 ± 13/40.5 ± 11	36.5 ± 12.5/38 ± 15.5
Percentile of TMT A (0–100)	48 ± 30/45.5 ± 25.5	52 ± 28/52 ± 29	53 ± 31/50 ± 24.5	61 ± 29/63 ± 25
Duration of TMT B (seconds)	96 ± 35/91 ± 34	83 ± 27/92 ± 31	85 ± 28.5/86.5 ± 32.5	81.5 ± 25/86 ± 34
Percentile of TMT B (0–100)	50 ± 24/54 ± 25	62.5 ± 23/54.5 ± 23	53 ± 27/58 ± 27	59 ± 24/60.5 ± 30
Score in Digit symbol subtest from WAIS-III (0–133)	59 ± 14/55.5 ± 16	60 ± 16/55.5 ± 11	59 ± 13.5/56.5 ± 11.5	62.5 ± 13.5/58 ± 13.5
Weighted score of Digit symbol subtest from WAIS-III (1–19)	11 ± 2.5/10.5 ± 3	11.5 ± 3/11 ± 2	11 ± 2.5/11 ± 2.5	12 ± 2.5/11 ± 2.5
Percentile of Digit symbol subtest from WAIS-III (0–100)	61 ± 26/53 ± 27.5	62 ± 26/58 ± 23	59 ± 24.5/62 ± 24	69.5 ± 22/60.5 ± 23

There were no significant differences in the neuropsychological tests between the two groups at every visit. Data are presented as mean ± standard deviation. Group PROPLA (A): probiotics first, placebo later; group PROPLA (B): placebo first, probiotics later; RAVLT, the Rey Auditory Verbal Learning Testp; TMT A and B, Trail Making Test parts A and B; ABACO, the Assessment BAttery of Cognition; PICNIR, the PICture Naming and Immediate Recall.

**Table 4:** Results of questionnaires and visual analog scales and their comparisons between the groups PROPLA and PLAPRO at several visits.

Visit	Visit 1 (plus Visit 2) baseline	Visit 3 (assessments after three months)	Visit 4 (assessments after three months)	Visit 5 (assessments after two months)
	Group PROPLA (A1)/group PLAPRO (B1)	Group PROPLA after probiotics (A3)/group PLAPRO after placebo (B3)	Group PROPLA after placebo (A4)/group PLAPRO after probiotics (B4)	Group PROPLA without intervention (A5)/group PLAPRO without intervention (B5)
Number of participants	40/32	37/30	37/30	16/15
Intervals from baseline assessments (days)	0	98 ± 10/96 ± 10	98 ± 20/96 ± 15	56 ± 11/57 ± 14
GDS total score (0–15 points)	2.5 ± 2/2.5 ± 1.5	2 ± 2/2 ± 2	2 ± 2.5/2 ± 2.5	2.5 ± 2.5/3 ± 3
BDI total score (0–63 points)	8 ± 4.5/7 ± 4.5	6 ± 5/6 ± 5	6.5 ± 6.5/6 ± 5	7.5 ± 7/6.5 ± 5.5
BAI total score (0–63 points)	5.5 ± 4.5/5 ± 4	5.5 ± 4.5/5 ± 4	5 ± 5/4 ± 4	5 ± 4/6 ± 4
FAQ total score (0–30 points)	1 ± 1.5/1 ± 2	1 ± 1.5/1.5 ± 2	1 ± 2/1 ± 1	1 ± 1.5/1.5 ± 1.5
Visual analog scale question 1 memory (0–10 points)	5 ± 2.5/5 ± 2	5.5 ± 2.5/5 ± 1.5	5.5 ± 3/5.5 ± 1.5	6 ± 2/4.5 ± 2.5
Visual analog scale question 2 digestion (0–10 points)	7.5 ± 2/8 ± 2	8 ± 1.5/7 ± 3	8 ± 1.5/8 ± 2	8 ± 1.5/7.5 ± 2.5
Visual analog scale question 3 overall health (0–10 points)	7 ± 2/7.5 ± 2	7.5 ± 1.5/7.5 ± 1.5	7 ± 2/8 ± 2	7 ± 1/7 ± 2.5
Visual analog scale question 4 sleep (0–10 points)	6 ± 2.5/6.5 ± 2.5	6 ± 2.5/6.5 ± 2	6.5 ± 3/7 ± 2	5.5 ± 2.5/6.5 ± 2.5
Visual analog scale question 5 feeling of anxiety (0–10 points)	1 ± 1.5/1 ± 1.5	1 ± 1.5/1 ± 1.5	1.5 ± 2/1.5 ± 2	1 ± 1/2 ± 2.5
Visual analog scale question 6 tiredness (0–10 points)	3 ± 2/3 ± 2	3 ± 2/3 ± 2	3.5 ± 2/3.5 ± 2	4 ± 2/3 ± 2.5
Visual analog scale question 7 pain (0–10 points)	2.5 ± 2.5/2 ± 2.5	2.5 ± 2.5/2 ± 2.5	2 ± 2/2 ± 2	2.5 ± 2.5/2.5 ± 2.5

There were no significant differences in the questionnaires and the visual analog scales between the two groups at any visit. Data are presented as mean ± standard deviation. Group PROPLA (A): probiotics first, placebo later; group PROPLA (B): placebo first, probiotics later; GDS, Geriatric depression scale; BDI, Beck depression inventory; BAI, Beck anxiety inventory; FAQ, the Functional Activities Questionnaire.

**Table 5:** Results of personal body characteristics and physical performances and their comparisons between the groups PROPLA and PLAPRO move this preposition to the new row several visits.

Visit	Visit 1 (plus Visit 2) baseline	Visit 3 (assessments after three months)	Visit 4 (assessments after three months)	Visit 5 (assessments after two months)
	Group PROPLA (A1)/group PLAPRO (B1)	Group PROPLA after probiotics (A3)/group PLAPRO after placebo (B3)	Group PROPLA after placebo (A4)/group PLAPRO after probiotics (B4)	Group PROPLA without intervention (A5)/group PLAPRO without intervention (B5)
Number of participants	40/32	37/30	37/30	16/15
Intervals from baseline assessments (days)	0	98 ± 10/96 ± 10	98 ± 20/96 ± 15	56 ± 11/57 ± 14
Weight (kg)	79 ± 20/78.5 ± 14.5	85 ± 22.5/79 ± 13.5	83 ± 21/79 ± 13.5	na
Body Mass Index (BMI)	28.5 ± 6/26.5 ± 3.5	29.5 ± 7/27 ± 3.5	29 ± 6.5/27 ± 3	na
Body water percentage	42 ± 5.5/41 ± 5	41 ± 5.5/42.5 ± 4.5	43.5 ± 7/44.5 ± 5	na
Muscle mass percentage	31 ± 3.5/30.5 ± 2.5	31 ± 3.5/32 ± 3	33 ± 5/32 ± 7	na
Bone weight (kg)	5 ± 1/5.5 ± 0.5	4.5 ± 1/4.5 ± 1	6 ± 6/5 ± 1	na
Number of lifting the one-kilogram dumbbell in 30 s	20.5 ± 5/21.5 ± 5.5	22 ± 4/22.5 ± 5.5	22.5 ± 4.5/24.5 ± 8	24.5 ± 5/25 ± 6.5
The time to walk 34 meters (seconds)	29 ± 4/27.5 ± 4	28 ± 7/27 ± 4	28 ± 5.5/27 ± 4.5	29.5 ± 4/25.5 ± 7.5
Number of repeated standing up and sitting on a chair in 30 s	12 ± 2/12.5 ± 3	12.5 ± 3/12.5 ± 3	12.5 ± 3/12.5 ± 2.5	13.5 ± 2.5/14 ± 3.5

There were no significant differences in all the measures between the two groups at any visit. Data are presented as mean ± standard deviation. Group PROPLA (A): probiotics first, placebo later; group PROPLA (B): placebo first, probiotics later; na, not available.

No differences in any measures were found between the two online groups (Supplementary Tables S2–S5) or the differences were due to very small numbers (Supplementary Table S5).

#### Longitudinal changes

Score differences in all the neuropsychological tests but one and the ALBA and the PICNIR did not change significantly in either group after the first 3 months and between examinations at 3 and 6 months. The only changes were observed for the TMT B test. They are shown alongside the results of two cognitive measures, RAVLT and ALBA, in Table 6 since the following text is rather complicated to read and understand.

**Table 6:** Longitudinal changes of selective cognitive tests in the groups PROPLA and PLAPRO and their comparisons.

Cognitive measure		The first interval of 3 months		The second interval of 3 months	
	Group	Change between visits 1 and 3	Interpretation	Change between visits 3 and 4	Interpretation
Duration of TMT B (seconds)	PROPLA	**−13 ± 20**	improvement after probiotics	2 ± 15	
	PLAPRO	**3 ± 25**		–5 ± 20	no change
	value of *p*	0.007		0.08	
Number of words on delayed recall in RAVLT (0–15 words)	PROPLA	0.9 ± 2		0.2 ± 2	
	PLAPRO	0.5 ± 2		0.7 ± 2	
	value of *p*	0.5	no change	0.3	no change
ALBA sentence recall – number of correctly recalled words of the sentence after distraction using the TEGEST (0–6 words)	PROPLA	**−1.8 ± 1.7**	worsening after probiotics	**2.2 ± 2**	improvement after placebo
	PLAPRO	**−0.6 ± 1.8**		**0.6 ± 1.9**	
	Value of *p*	0.006		0.001	

Data are presented as mean ± standard deviation. Group PROPLA (A): probiotics first, placebo later; group PROPLA (B): placebo first, probiotics later; TMT B, Trail Making Test part B; RAVLT, the Rey Auditory Verbal Learning Test; ALBA, the Amnesia Light and Brief Assessment test. Statistically significant changes are highlighted in bold.

The time to complete TMT B reduced from baseline visit 1 to visit 3 (−13 ± 20 s in group A PROPLA vs. 3 ± 25 s in group B PLAPRO; *p* = 0.007), i.e., a favorable effect of probiotics over placebo. However, it did not significantly change between visits 3 and 4 (2 ± 15 s in group A PROPLA vs. −5 ± 20 s in group B PLAPRO; *p* = 0.08), although the trend was the same (time reduction after probiotic use and increase after placebo).

The most important memory outcome was the number of words in delayed recall in the RAVLT. Participants in both groups improved a little and without statistical significance after 3 months (0.9 ± 2 in group A PROPLA vs. 0.5 ± 2 in group B PLAPRO; *p* = 0.5) and between 3 and 6 months (0.2 ± 2 in group A PROPLA vs. 0.7 ± 2 in group B PLAPRO; *p* = 0.3).

The number of correctly recalled words of the sentence after distraction in the ALBA test showed cross-sectional changes (Table 2). This ALBA sentence recall significantly varied after 3 months (−1.8 ± 1.7 in group A PROPLA vs. −0.6 ± 1.8 in group B PLAPRO; *p* = 0.006, i.e., worsening after probiotics) and between 3 and 6 months (2.2 ± 2 in group A PROPLA vs. 0.6 ± 1.9 in group B PLAPRO; *p* = 0.001, i.e., smaller increase after probiotics).

We did not find any influence of age and gender on changes in RAVLT score and TMT B time in either group PROPLA/PLAPRO after the first and the second 3 months.

### Safety evaluation

The counts of specific symptoms reported during each visit are presented in Supplementary Table S6. The most commonly reported symptoms were insomnia in Group A (16%) and constipation, allergies, and insomnia in Group B (10% each) at baseline visit 1. Constipation improved after probiotics whereas it got worse after placebo at visit 3. This ratio also remained at visit 4.

### Participant drop-out

Only seven participants decided to withdraw from the study. Thus, dropout rate was low [7/(72 in person +19 online) = 8%]. One stopped after Visit 1 because of a ruptured hemorrhoid. Five discontinued between Visit 2 and Visit 3 due to discomfort experienced during the blood sampling (*n* = 1), persistent gastrointestinal problems experienced during the treatment or placebo consumption (*n* = 3), and unwillingness to continue in the study (*n* = 1). One participant withdrew after Visit 3 due to an operation and subsequent hospital stay. The reasons for withdrawal and group assignment are summarized in Supplementary Table S7.

## Discussion

This is the longest randomized clinical trial with a cross-over design conducted in Europe to evaluate the effects of human probiotics on memory and psychological and physical measures using the comprehensive approach. Community-dwelling older adults were recruited and selected using modern digital technology with a sophisticated electronic questionnaire and the memory test ALBAV (Bartos and Krejcova, 2022; Bartos and Krejcova, 2023). This distance assessment and pre-screening were cost-effective since the initial 724 individuals interested in our probiotic study were reduced to 91 (13%) eligible participants only, i.e., the ratio of 8 interested individuals: 1 individual included in the study. Participants were randomized into two groups matched for sociodemographic and psychological measures at baseline. All were followed for 6 months and some of them for 8 months. The effects of probiotics were measured twice in each group independently due to the cross-over design which would confirm the consistency of observations. In contrast to our hypothesis, we did not find changes in basically any of the measures in any group. We monitored the status of the participants using an extensive range of measures. Brief cognitive tests were included to verify whether such methods could identify the impact of probiotics in future studies. However, even standard neuropsychological tests did not show a positive response to the probiotic intervention. Neuropsychological tests did not provide an added value to prove the probiotic effect. The results of questionnaires and visual analog scales of depression, anxiety, and related symptoms did not change throughout the whole study. Personal body characteristics and physical performances also remained the same. Altogether, we did not observe favorable effects of novel human probiotics on cognitive, affective, and physical functions.

There are a number of possible explanations for our results. The effects of probiotic ingestion may be influenced by the host’s side and by the probiotic’s side (Białecka-Dębek et al., 2021). Host factors include the level of cognitive functioning, diet and lifestyle, age, sex, geographic region, comorbid disease, antibiotic exposure, and baseline microbiota composition and these factors should be controlled. Differences in probiotic strains, dosage, and intervention duration can also modify the outcomes (Białecka-Dębek et al., 2021).

Probiotics themselves are also an important factor in the efficiency assessment. The strains chosen for our trial might not have been right for this purpose despite their human origin and thus assumed better efficacy. The dose of probiotics might have been low (10^6^ colony-forming units). The usual dosage in other studies is 10^8^–10^11^ colony-forming units (Białecka-Dębek et al., 2021). The duration of 3 months might have been short, though this is the most common duration in similar studies (Białecka-Dębek et al., 2021). Long-term studies would be desirable, but it is difficult to organize them within the usual 3- to 4-year grant. The cross-over design doubles the time to the end of the trial, e.g., a 9-month probiotic intervention requires 18 months of the trial. Longer trials are needed from a scientific point of view since the response to probiotics may be delayed in gut microbiota and subsequently in brain functions. However, the longer the duration of any design the more confounding factors and drop-outs which may limit conclusions. In addition, it will be more costly and time-consuming, and it will require personnel stability. On the contrary, one meta-analysis showed a contra-intuitive finding that a duration of less than 3 months of probiotics was more effective (Lv et al., 2021). In line with our outcomes, a recent systematic review of studies exploring the effects of probiotics on memory or mood in elderly individuals did not find positive effects of probiotics on cognition (Tahmasbi et al., 2022). Some previous studies also showed that probiotic use did not improve cognitive function or mood (Benton et al., 2007; Chung et al., 2014; Ohsawa et al., 2018; Louzada and Ribeiro, 2020).

In one of the studies, either a probiotic-containing milk drink or a placebo were consumed daily for a 3-week period in a UK sample. Those who had consumed the milk drink had significantly worse memory scores (Benton et al., 2007). We also observed a similar unexpected finding in our clinical trial. For participants in the PROPLA group, consuming probiotics for 3 months resulted in slightly poorer performance on ALBA sentence recall. This is possibly a chance result since similar worsening was not observed in the second 3 months in the PLAPRO group (Table 2). The verbal fluency scores of the previous study did not change, similar to our results. Similarly to our results, the consumption of the probiotic did not change the mood in the Benton et al. (2007) study due to the overall good mood measured in their sample.

Negative results after probiotic use were also reported in healthy individuals in Asia and Brazil. No change or similar improvement was observed in placebo and probiotic groups (Chung et al., 2014; Inoue et al., 2018; Ohsawa et al., 2018; Kobayashi et al., 2019; Louzada and Ribeiro, 2020).

Some previous studies also show positive effects of probiotics. Supplementation of healthy middle-aged adults with a *L. helveticus*-fermented milk drink for 8 weeks improved both attention and delayed memory subscores of the Repeatable Battery for the Assessment of Neuropsychological Status. However, these changes were statistically significant only for comparisons between values before vs. after intake. When values were compared after intake vs. placebo group, the significance for delayed memory disappeared and it was preserved for attention only. It is questionable whether differences of 1–5 points out of 56 for attention or 1–3 points out of 42 for delayed memory (i.e., 5% on average) (Ohsawa et al., 2018) represent a clinically meaningful effect.

As we explained earlier in our protocol paper (Bartos et al., 2022), people with mild cognitive deficits would be the appropriate target for probiotic influence. The general cognitive abilities of our participants were around the 50th percentile, i.e., the average (Table 3). During the electronic recruitment we selected only those participants who were in the worst third of scores in the electronic ALBAV test. If we had not done this our sample would likely include individuals with even better cognitive function. This would make it even more difficult to prove cognitive improvement, i.e., high scores could not be higher. We excluded 356 candidates with better scores in the electronic test ALBAV. Examining all these candidates or even all the individuals meeting eligibility criteria in person (*n* = 724-232-45 = 447) (see Figure 2 in our previous protocol paper Bartos et al., 2022) would be ineffective and probably impossible due to time constraints. Moreover, to avoid learning effects the cognitive tests in such pre-testing would have to have been different to those in the clinical trial itself. Such a strategy would also be costly for participants as they would have to visit the testing center for five (instead of four) examinations within 9–18 months which could lead to lower adherence to the study. It would also increase logistic demands. Due to these reasons, it is unlikely we would be able to get such a large sample size if we used an in-person instead of the electronic screening approach. In summary, electronic recruitment and cognitive screening have proven to be beneficial and we recommend this approach if feasible to reduce the workload and time to organize such studies as well as to ensure sufficient sample size.

Another recruitment method could have been to include patients from memory clinics and not community-dwelling older adults. The advantage of this approach is that their cognitive status would be known from past diagnosis. We have considered recruiting such patients. However, it has many drawbacks which cannot be easily solved.

First, patients with mild cognitive impairment are rare in our memory clinic based on our long-term experience. Even in individuals who visit our memory clinic at early stages of cognitive impairment we frequently find subtle or mild impairment of activities of daily living and thus they fulfill criteria for mild dementia rather than for MCI (American Psychiatric Association, 2015). This is in agreement with a systematic review that found deficits in instrumental activities of daily living even in patients diagnosed with MCI (Jekel et al., 2015). This poses a problem for screening a sufficient number of individuals in a reasonable period.

Second, patients described above and intended for probiotic studies of cognition should be free of gastrointestinal, psychiatric, and neurological brain disorders or other confounding comorbidities that may interfere with probiotic intake and digestion or their cognitive functions (coeliac disease, ulcerative colitis, stroke, schizophrenia, heart, hepatic insufficiency, dysimmunity, oncological diseases or treatment, operations involving general anesthesia, probiotic or antibiotic use, etc.). This would further reduce eligible candidates from a small number of patients.

Third, participants must have good sight and hearing for cognitive testing, they must agree to come and be examined several times (4–5 times in our trial), agree to take pills regularly every day, and adhere to the study for a period of several months (6–8 months in our trial). Moreover, in our trial, participants had to be able to collect their own stool and urine samples. Overall, these factors made our recruitment method most efficient and allowed us to have a larger sample than is typical for clinical studies of such length in this area. While the first drawback described specifically refers to memory clinics outpatients, it seems that the other issues listed can also be applied to the general population.

Our trial has several strengths. These include the electronic recruitment and selection, exact baseline matching of both groups, homogenous population due to highly stringent inclusion and exclusion criteria and selection, allocation concealment, blinding of participants and personnel, longer duration and double evaluations in the cross-over design in two groups of reasonable sizes, comprehensive evaluations involving brief and neuropsychological cognitive tests, several mood and self-report questionnaires, personal physical characteristics and performance measures, additional blood, urine, and stool sampling, online assessments in a subset due to COVID-19 pandemic, the use of alternative versions in repeated examinations, measurement of apolipoprotein E status, and simultaneous intake of probiotics and prebiotics.

This study has some limitations. First, since the participants took tablets at home one cannot be sure about their compliance. We assessed it retrospectively during visits 3 and 4. Nobody confessed to not taking study medication. There is no way to find out whether they took tablets correctly. This is a general problem of oral use of tablets at home either under study conditions or for own treatment when treatment is not personally overseen by the researchers or someone else. However, we assume high adherence of participants to tablet use because they were highly motivated with a belief in improving self-health. Second, the apolipoprotein E type 4 allele occurred in too few individuals to allow any analysis of whether this factor may influence probiotic efficiency. Third, other dosages of probiotics, strains of flora, the proportion of each strain, and total intervention duration could yield different results (Białecka-Dębek et al., 2021). Additionally, our sample was extremely homogenous due to the many eligibility criteria and therefore not representative of the general population within the same age range.

The negative results of our clinical trial mean that probiotic intervention in general is not the right approach to enhance cognitive functions or that future clinical trials should be better arranged to achieve beneficial outcomes. Now we will propose some amendments that might be addressed in the design of future interventions. The first are related to probiotics and the second to individuals themselves (Białecka-Dębek et al., 2021).

The first factors include prebiotics and strains, dose, and duration of probiotics. It is important to explore and identify the type of prebiotics which promote probiotic growth best. Prebiotics are nutrition for probiotics and thus are also important for their activities and metabolism. It is not clear whether single- or multiple-strain probiotics are more useful. Some strains may have a positive effect on cognitive function, while others may not have psychobiotic potential. An appropriate dose of probiotics is unknown. Doses often ranged from 10^8^ to 10^11^. The use of higher doses beyond these conventional doses should be considered (Białecka-Dębek et al., 2021; Lv et al., 2021; Xiang et al., 2022). Direct comparisons of strains and doses would provide new insights for further development in this field. The duration of probiotic use has been discussed earlier. It is not about the intervention period itself but also an effect following the end of the intervention whether it will persist after the study termination and for how long. It was evaluated in an extended follow-up after termination of our clinical trial without any difference when compared to the last examination. We propose to plan such a follow-up assessment in study preparation.

The second factors are related to participants regarding their recruitment, selection, cognitive functions, and assessments. We recommend screening people via electronic questionnaires with questions of inclusion/exclusion criteria and via some computerized test, e.g., the ALBAV in our study (Bartos and Krejcova, 2022; Bartos and Krejcova, 2023). It results in a relatively homogenous sample without certain comorbidities. The appropriate target population includes individuals with mild cognitive deficits. Patients with dementia have advanced and irreversible brain pathology. By contrast, healthy people with normal baseline cognitive functions cannot improve them furthermore due to the ceiling effect.

The effect of probiotics can be monitored using several comprehensive sets of cognitive tests, subjective questionnaires, visual scales, physical fitness tests, stool samples, etc. Adverse effects should also be recorded. Changes in cognitive functions can be assessed with both brief tests and a neuropsychological battery to show whether the influence of probiotics can be demonstrated with either approach or both. If proven, physicians and other professionals could utilize brief instruments in their practice to verify a favorable effect of probiotics. It is advisable to analyze baseline stool samples or microbiota to incorporate this information into personalized management in the future.

In conclusion, our probiotics with prebiotics did not improve cognitive, affective, or physical measures in community-dwelling older adults selected using stringent eligibility criteria. It is another piece of evidence along with previous failures that this approach might not provide a benefit to cognitive functioning of older adults (Tahmasbi et al., 2022). A similar study also using a crossover design is ongoing to assess the efficacy of a multispecies probiotic formulation. This therapeutic strategy aims to improve the emotional and cognitive decline associated with aging in adults over the age of 55 years (Ruiz-Gonzalez et al., 2022). It will be interesting to see whether their outcomes will be in the same direction as ours.

## Data availability statement

The raw data supporting the conclusions of this article will be made available by the corresponding author, without undue reservation.

## Ethics statement

The studies involving human participants were reviewed and approved by Ethics Committee of the National Institute of Mental Health, Klecany Ethics Committee of the University Hospital Kralovske Vinohrady. The patients/participants provided their written informed consent to participate in this study.

## Author contributions

AB: conceptualization and design of the study, data analysis and interpretation, table outlining, writing of an original manuscript, and funding acquisition. JW: collection and analysis of the data, drafting the method, revision of the manuscript, and preparation of the figure. SD: preparation of a majority of the tables and review and editing of the manuscript. All authors contributed to the article and approved the final manuscript.

## Funding

This study was supported by the program Trio [FV40032] (CleverAge Biota) from the Ministry of Industry and Trade, program of Ministry of Health, Czech Republic – conceptual development of research organization (FNKV, 00064173), and the program Cooperatio, Neuroscience Charles University.

## Conflict of interest

The authors declare that the research was conducted in the absence of any commercial or financial relationships that could be construed as a potential conflict of interest.

## Publisher’s note

All claims expressed in this article are solely those of the authors and do not necessarily represent those of their affiliated organizations, or those of the publisher, the editors and the reviewers. Any product that may be evaluated in this article, or claim that may be made by its manufacturer, is not guaranteed or endorsed by the publisher.

## Supplementary material

The Supplementary material for this article can be found online at: https://www.frontiersin.org/articles/10.3389/fnagi.2023.1163727/full#supplementary-material

## References

[ref1] American Psychiatric Association (2015). “Diagnostic and statistical manual of mental disorders (DSM-5®)” in book in Czech: Diagnostický a statistický manuál duševních poruch. 5th ed (Praha: Hogrefe – Testcentrum), 633–675.

[ref2] Bartos A. (2016). Do not test but PICNIR (ENTERTAIN) - written intentional naming of pictures and their recall as a brief cognitive test [article in Czech Netestuj, ale POBAV - písemné záměrné pojmenování obrázků a jejich vybavení jako krátká kognitivní zkouška]. Cesk. Slov. Neurol. N. 79, 671–679.

[ref3] Bartos A. (2019). Two original Czech tests for memory evaluation in three minutes – amnesia light and brief assessment (ALBA) [article in Czech Dvě původní české zkoušky k vyšetření paměti za tři minuty – amnesia light and brief assessment (ALBA)]. Cesk. Slov. Neurol. N. 82, 420–429. doi: 10.14735/amcsnn2019420

[ref4] Bartos A. (2021). ALBA and PICNIR tests used for simultaneous examination of two patients with dementia and their adult children. Cesk. Slov. Neurol. 84/117, 583–586. doi: 10.48095/cccsnn2021583

[ref5] Bartos A. Diondet S. (2020). Amnesia light and brief assessment (ALBA) test – the second version and repeated examinations [article in Czech test amnesia light and brief assessment (ALBA) – druhá verze a opakovaná vyšetření]. Cesk. Slov. Neurol. N. 82, 535–543. doi: 10.14735/amcsnn2020535

[ref6] Bartos A. Diondet S. (2023). Sensitive amnesia light and brief assessment (ALBA) is a valid three-minute test of four tasks indicative of mild cognitive deficits. Neurologia (in press). 10.1016/j.nrleng.2025.06.012 40769607

[ref7] Bartos A. Janoušek M. Petroušová R. Hohinová M. (2016). Three times of the clock drawing test rated with BaJa scoring in patients with early Alzheimer’s disease [article in Czech Tři časy Testu kreslení hodin hodnocené BaJa skórováním u časné Alzheimerovy nemoci]. Cesk. Slov. Neurol. N. 112, 406–412.

[ref8] Bartos A. Krejcova M. (2022). Development of the electronic memory test for the elderly (ALBAV) [article in Czech Vývoj elektronického testu paměti pro starší osoby (ALBAV)]. Cesk. Slov. Neurol. N. 85, 369–374. doi: 10.48095/cccsnn2022369

[ref9] Bartos A. Krejcova M. (2023). Validation of the electronic memory test ALBAV [article in Czech Validizace elektronického testu paměti ALBAV]. Cesk. Slov. Neurol. N. 86/119, 49–56. doi: 10.48095/cccsnn202349

[ref10] Bartos A. Polanská H. (2021). Correct and incorrect naming of pictures for the more demanding written Picture naming and immediate recall test (door PICNIR) [article in Czech Správná a chybná pojmenování obrázků pro náročnější test písemného Pojmenování obrázků a jejich vybavení (dveřní POBAV)]. Cesk. Slov. Neurol. N. 84, 151–163. doi: 10.48095/cccsnn2021151

[ref11] Bartos A. Weinerova J. Diondet S. Vales K. (2022). Effect of human probiotics on memory, psychological and biological measures in elderly: a study protocol of bi-center, double-blind, randomized, placebo-controlled clinical trial (CleverAge biota). Front. Aging Neurosci. 14:996234. doi: 10.3389/fnagi.2022.996234 36437993 PMC9686296

[ref12] Beck A. T. Epstein N. Brown G. Steer R. A. (1988). An inventory for measuring clinical anxiety: psychometric properties. J. Consult. Clin. Psychol. 56, 893–897. doi: 10.1037//0022-006x.56.6.893 3204199

[ref13] Beck A. T. Steer R. A. Brown G. K. (1996). Beck depression inventory (BDI-II), 10. London: Pearson.

[ref14] Benton D. Williams C. Brown A. (2007). Impact of consuming a milk drink containing a probiotic on mood and cognition. Eur. J. Clin. Nutr. 61, 355–361. doi: 10.1038/sj.ejcn.1602546 17151594

[ref15] Białecka-Dębek A. Granda D. Szmidt M. K. Zielińska D. (2021). Gut microbiota, probiotic interventions, and cognitive function in the elderly: a review of current knowledge. Nutrients 13:2514. doi: 10.3390/nu13082514 34444674 PMC8401879

[ref16] Bridel C. van Wieringen W. N. Zetterberg H. Tijms B. M. Teunissen C. E. the NFL Group. (2019). Diagnostic value of cerebrospinal fluid neurofilament light protein in neurology: a systematic review and meta-analysis. JAMA Neurol. 76, 1035–1048. doi: 10.1001/jamaneurol.2019.1534 31206160 PMC6580449

[ref17] Chung Y.-C. Jin H.-M. Cui Y.-K. Dal J. Jin P. Jong-Li J.. (2014). Fermented milk of *lactobacillus helveticus* IDCC3801 improves cognitive functioning during cognitive fatigue tests in healthy older adults. J. Funct. Foods 10, 465–474. doi: 10.1016/j.jff.2014.07.007

[ref18] Dvořáková T. Bušková J. Bartoš A. (2022). Neurological symptoms associated with COVID-19 based on a nation-wide online survey [article in Czech Neurologické příznaky asociované s onemocněním COVID-19 podle celostátního online průzkumu]. Cesk. Slov. Neurol. N. 85, 220–227. doi: 10.48095/cccsnn2022220

[ref19] Fialová L. Nosková L. Kalousová M. Zima T. Uher T. Bartoš A. (2022). Analytical and pre-analytical aspects of neurofilament light chain determination in biological fluids [article in Czech Analytické a preanalytické aspekty stanovení lehkých řetězců neurofilament v biologických tekutinách]. Cesk. Slov. Neurol. N. 85, 11–16. doi: 10.48095/cccsnn202211

[ref20] Inoue T. Kobayashi Y. Mori N. Sakagawa M. Xiao J.-Z. Moritani T.. (2018). Effect of combined bifidobacteria supplementation and resistance training on cognitive function, body composition and bowel habits of healthy elderly subjects. Benef. Microbes. 9, 843–853. doi: 10.3920/BM2017.0193 30198326

[ref21] Jekel K. Damian M. Wattmo C. Hausner L. Bullock R. Connelly P. J.. (2015). Mild cognitive impairment and deficits in instrumental activities of daily living: a systematic review. Alzheimers Res. Ther. 7:17. doi: 10.1186/s13195-015-0099-0 25815063 PMC4374414

[ref22] Kobayashi Y. Kuhara T. Oki M. Xiao J. (2019). Effects of *bifidobacterium breve* a1 on the cognitive function of older adults with memory complaints: a randomised, double-blind, placebo-controlled trial. Benef. Microbes. 10, 511–520. doi: 10.3920/BM2018.0170 31090457

[ref23] Krüger J. F. Hillesheim E. Pereira A. C. S. N. Camargo C. Q. Rabito E. I. (2021). Probiotics for dementia: a systematic review and meta-analysis of randomized controlled trials. Nutr. Rev. 79, 160–170. doi: 10.1093/nutrit/nuaa037 32556236

[ref24] Lezak M. D. Howieson D. B. Bigler E. D. Tranel D. (2012). Neuropsychological assessment. New York, NY: Oxford University Press.

[ref25] Louzada E. R. Ribeiro S. M. L. (2020). Synbiotic supplementation, systemic inflammation, and symptoms of brain disorders in elders: a secondary study from a randomized clinical trial. Nutr. Neurosci. 23, 93–100. doi: 10.1080/1028415X.2018.1477349 29788823

[ref26] Lv T. Ye M. Luo F. Hu B. Wang A. Chen J.. (2021). Probiotics treatment improves cognitive impairment in patients and animals: a systematic review and meta-analysis. Neurosci. Biobehav. Rev. 120, 159–172. doi: 10.1016/j.neubiorev.2020.10.027 33157148

[ref27] Ohsawa K. Nakamura F. Uchida N. Mizuno S. Yokogoshi H. (2018). *Lactobacillus helveticus*-fermented milk containing lactononadecapeptide (NIPPLTQTPVVVPPFLQPE) improves cognitive function in healthy middle-aged adults: a randomised, double-blind, placebo-controlled trial. Int. J. Food Sci. Nutr. 69, 369–376. doi: 10.1080/09637486.2017.1365824 28819993

[ref28] Pfeffer R. I. Kurosaki T. T. Harrah C. Jr. Chance J. M. Filos S. (1982). Measurement of functional activities in older adults in the community. J. Gerontol. 37, 323–329. doi: 10.1093/geronj/37.3.323 7069156

[ref29] Polanská H. Bartoš A. (2022). Telemedicine assessments by remote versions of ALBA, POBAV and ACE-III tests [article in Czech Telemedicínské vyšetření kognitivními testy ALBA, POBAV a ACE-III]. Cesk. Slov. Neurol. N. 85, 296–305. doi: 10.48095/cccsnn2022296

[ref30] Preiss M. Bartos A. Čermáková R. Nondek M. Benešová M. Rodriguez M.. (2012). “Neuropsychological battery of the psychiatric Prague center: clinical examinations of major cognitive functions” in A book in Czech: Neuropsychologická baterie Psychiatrického centra Praha: Klinické vyšetření základních kognitivních funkcí (3). 3rd ed (Praha: Psychiatrické centrum Praha)

[ref31] Ruiz-Gonzalez C. Cardona D. Rodriguez-Arrastia M. Ropero-Padilla C. Rueda-Ruzafa L. Carvajal F.. (2022). Effects of probiotics on cognitive and emotional functions in healthy older adults: protocol for a double-blind randomized placebo-controlled crossover trial. Res. Nurs. Health 45, 274–286. doi: 10.1002/nur.22209 35080033

[ref32] Sánchez-de-Lara-Sánchez S. Sánchez-Pérez A. M. (2022). Probiotics treatment can improve cognition in patients with mild cognitive impairment: a systematic review. J. Alzheimers Dis. 89, 1173–1191. doi: 10.3233/JAD-220615 36093709

[ref33] Tahmasbi F. Mirghafourvand M. Shamekh A. Mahmoodpoor A. Sanaie S. (2022). Effects of probiotic supplementation on cognitive function in elderly: a systematic review and Meta-analysis. Aging Ment. Health 26, 1778–1786. doi: 10.1080/13607863.2021.1966743 34428991

[ref34] Trejo-Castro A. I. Carrion-Alvarez D. Martinez-Torteya A. Rangel-Escareño C. (2022). A bibliometric review on gut microbiome and Alzheimer's disease between 2012 and 2021. Front. Aging Neurosci. 14:804177. doi: 10.3389/fnagi.2022.804177 35898324 PMC9309471

[ref35] Wallace C. J. K. Milev R. (2017). The effects of probiotics on depressive symptoms in humans: a systematic review. Ann. General Psychiatry 16:14. doi: 10.1186/s12991-017-0138-2 PMC5319175 28239408

[ref36] Xiang S. Ji J. L. Li S. Cao X. P. Xu W. Tan L.. (2022). Efficacy and safety of probiotics for the treatment of Alzheimer's disease, mild cognitive impairment, and Parkinson's disease: a systematic review and meta-analysis. Front. Aging Neurosci. 14:730036. doi: 10.3389/fnagi.2022.730036 35185522 PMC8851038

[ref37] Yesavage J. A. Brink T. L. Rose T. L. Lum O. Huang V. Adey M.. (1982). Development and validation of a geriatric depression screening scale: a preliminary report. J. Psychiatr. Res. 17, 37–49. doi: 10.1016/0022-3956(82)90033-4 7183759

