# Supplementary material for: Effects of human probiotics on memory and psychological and physical measures in community-dwelling older adults with normal and mildly impaired cognition: results of a bi-center, double-blind, randomized, and placebo-controlled clinical trial (CleverAge biota)
Source: Front Aging Neurosci. 2023 Jul 7;15:1163727. doi: 10.3389/fnagi.2023.1163727 (PMC10369778; doi:10.3389/fnagi.2023.1163727)
Supplement: Supplementary file 1 [file Table_1.docx]

**Supplementary** **Table 1 Online participant characteristics and comparisons of socio-demographically matched groups**

|  | **group PROPLA (A)** | **group PLAPRO (B)** | **p-value** |
| --- | --- | --- | --- |
| **Number of participants** | 7 | 12 |  |
| **Age** (years) | 64±5.5 | 68±7 | n.s. |
| **Education category** | 3 (43%)/ 4 (57%) | 2 (25%)/ 3 (25%)/ 4 (50%) | n.s. |
| **Education** (years of schooling) | 16.5±3 | 16±4 | n.s. |
| **Female number** (percent) | 5 (71%) | 9 (75%) | n.s. |
| **Height (cm)** | 168±12 | 169±10 | n.s. |
| **Weight (kg)** | 84.5±21 | 69±13 | n.s. |
| **BMI** | 28.5±5.5 | 24.5±4.5 | n.s. |
| **Hearing abilities** (percent) | 100% | 100% | n.s. |
| **Visual abilities** (percent) | 100% | 100% | n.s. |

group PROPLA (A): probiotics first, placebo later; group PROPLA (B): placebo first, probiotics later; education (category): 1 = primary school, 2 = high school without a General Certificate of Education (GCE), 3 = high school with GCE, 4 = university, BMI – body mass index, GDS – Geriatric Depression Scale, p – probability, n.s. – not significant. Data are presented as mean ± standard deviation.

**Supplementary Table 2 Results of online brief cognitive tests and their comparisons between the groups PROPLA and PLAPRO at several times**

The differences of all the brief cognitive tests were not significant between both groups at all times except of those in bold and with asterisks.

|  | **group PROPLA (A1) /** **group PLAPRO (B1)** | **group PROPLA after probiotics (A3)/** **group PLAPRO after placebo (B3)** | **group PROPLA after placebo (A4)/** **group PLAPRO after probiotics (B4)** |
| --- | --- | --- | --- |
| **Visit** | **Visit 1 (plus Visit 2)**  **baseline** | **Visit 3 (assessments after three months)** | **Visit 4 (assessments after three months)** |
| **Number of participants** | 7/12 | 7/11 | 7/9 |
| **Intervals from baseline assessments (days)** | 0 | 94±7/ 91±7 | 91±2/ 96±8 |
| **ALBA Sentence encoding**- number of correctly repeated words of the sentence (0-6 words) | 5.5±0.5/ 5±1 | 5.5±1/ 5.5±1 | 5.5±0.5/ 5.5±1 |
| **ALBA Sentence recall** – number of correctly recalled words of the sentence after distraction using the TEGEST (0-6 words) | 4.5±2/ 4±2 | 5±1.5/ 3.5±2 | 5±1/ 4.5±2 |
| **ALBA TEGEST** – initial demonstration of six gestures according to instructions (0-6 gestures) | 6±0/ 6±0 | 6±0/ 6±0 | 6±0 / 6±0 |
| **ALBA TEGEST gesture recall** – number of correctly recalled gestures (0-6 gestures) | 4.5±1/ 4.5±1.5 | 4.5±0.5/ 4.5±1 | 4.5±1/ 4±1 |
| **ALBA memory score–** the sum of the number of correctly recalled sentence words and gestures (0-12 points) | 9.5±2/ 8.5±2.3 | 9.5±1.5/ 8±2.5 | 10±1/ 9±3 |
| **ABACO test version** | version 1 | version 2 | version 1 |
| **ABACO Reading Encrypted Sentences subtest** (0-3 points) | 2.5±1/ 2.5±0.5 | 2.5±1/ 2.5±0.5 | 3±0.5/ 2.5±0.5 |
| **ABACO** **sentence learning/ encoding first trial (**0-10 words) | 8±2.5/ 7±2 | 6.5±1.5/ 6.5±1.5 | 8.5±1/ 7.5±2 |
| **ABACO** **sentence learning/encoding second trial (**0-10 words) | 9.5±0.5/ 8.5±1.5 | 8.5±1/ 8.5±1.5 | 9.5±1/ 9±1.5 |
| **ABACO sentence immediate recall** (0-10 words) | 9±1/ 8±2 | 8±1.5/ 7±2 | 9.5±1/ 7±3 |
| **ABACO sentence delayed recall** (0-10 words) | **8±1.5/ 5±3* (7.8 vs 5.2) (p=0.03)** | 7.5±2/ 5.5±2.5 | 8.5±1/ 5.5±3.5*** (8.7 vs 5.5) (p=0.04)** |
| **ABACO verbal fluence task in 30 seconds** | 12.5±2.5/ 13.5±4 | 11.5±3.5/ 12.5±4.5 | 13.5±2/ 11.5±3 |
| **ABACO the PICNIR Picture naming mistakes** – number of mistakes or unnamed pictures (0-20 pictures) | 0.5±0.5/ 0±0.5 | 0.5±1/ 1±0.5 | 0.5±0.5/ 0.5±0.5 |
| **ABACO the PICNIR Picture naming recall** – number of correctly recalled pictures (0-20 words) | 9.5±3/ 7.5±3 | 10.5±2/ 8±3.5 | 11±1/ 9±3 |
| **ABACO the Four or the Five-line test score** (0-4 points) | 3.5±1/ 3.5±1 | 4±0/ 3.5±1 | 4±0/ 3±1.5 |
| **ABACO total score** (0-35 points) | 29±1/ 24±7 | 29±2/ 24.5±8 | 32.5±2.5/ 25.5±8.5*** (32.7 vs 25.3) (p=0.04)** |

group PROPLA (A): probiotics first, placebo later; group PROPLA (B): placebo first, probiotics later, CDT – the Clock Drawing Test, BaJa – scoring by Bartos and Janousek, ALBA – the Amnesia Light and Brief Assessment test, TEGEST – the test of gestures, RAVLT - the Rey Auditory Verbal Learning Test, TMT A and B - Trail Making Test parts A and B, ABACO - the Assessment BAttery of Cognition, PICNIR – the PICture Naming and Immediate Recall, n.s. – not significant. Data are presented as mean ± standard deviation, p- values show probabilities (probiotics versus placebo)

**Supplementary Table 3 Results of online neuropsychological tests and their comparisons between the groups PROPLA and PLAPRO at several visits**

The differences of all the neuropsychological tests were not significant between both groups at all visits.

|  | **group PROPLA (A1) /** **group PLAPRO (B1)** | **group PROPLA after probiotics (A3)/** **group PLAPRO after placebo (B3)** | **group PROPLA after placebo (A4)/** **group PLAPRO after probiotics (B4)** |
| --- | --- | --- | --- |
| **Visit** | **Visit 1 (plus Visit 2)**  **baseline** | **Visit 3 (assessments after three months)** | **Visit 4 (assessments after three months)** |
| **Number of participants** | 7/12 | 7/11 | 7/9 |
| **Intervals from baseline assessments** (days) | 0 | 94±7/ 91±7 | 91±2/ 96±8 |
| **Score of Category Fluency Test** (animals in one minute) | 28±5.5/ 25.5±9 | 29.5±3.5/ 26±8 | 28.5±5/ 24.5±9 |
| **RAVLT test version** | version 1 | version 2 | version 3 |
| **Total number of words in sets A 1-5 in RAVLT** (0-75 words) | 52±4/  46.5±12.5 | 54±7/  48±13 | 55.5±8/ 48±14.5 |
| **Percentile in sets A 1-5 in RAVLT** (0-100) | 53.5±17.5/  47*±*30.5 | 50.5±23.5/ 52±26.5 | 50±0/  51.5±36.5 |
| **Number of words on delayed recall in RAVLT** (0-15 words) | 9±4/ 9.5±4 | 9.5±3/ 9.5±5 | 11.5±2.5/ 9±5.5 |
| **Percentile of delayed recall in RAVLT** (0-100) | 42±27.5/  50.5±29.5 | 46.5±37.5/ 56.5±34 | 40±0/ 50±36.5 |
| **Score of Phonemic Fluency Test in three minutes** (initial letters NKP/VOZ/NKP per one minute) | 46.5±14/ 44±14 | 44±9.5/ 40.5±10 | 52±11.5/ 45±14.5 |

group PROPLA (A): probiotics first, placebo later; group PROPLA (B): placebo first, probiotics later, RAVLT - the Rey Auditory Verbal Learning Test, TMT A and B - Trail Making Test parts A and B, ABACO - The Assessment BAttery of Cognition, PICNIR – the PICture Naming and Immediate Recall. Data are presented as mean ± standard deviation

**Supplementary Table 4 Results of questionnaires and visual analogue scales and their comparisons between the online groups PROPLA and PLAPRO at several visits**

The differences of all the scores of the questionnaires and the visual analogue scales were not significant between both groups at all visits except of those in bold and with asterisks.

|  | **group PROPLA (A1) /** **group PLAPRO (B1)** | **group PROPLA after probiotics (A3)/** **group PLAPRO after placebo (B3)** | **group PROPLA after placebo (A4)/** **group PLAPRO after probiotics (B4)** |
| --- | --- | --- | --- |
| **Visit** | **Visit 1 (plus Visit 2)**  **baseline** | **Visit 3 (assessments after three months)** | **Visit 4 (assessments after three months)** |
| **Number of participants** | 7/12 | 5/10 | 6/8 |
| **Intervals from baseline assessments** (days) | 0 | 94±7/ 91±7 | 91±2/ 96±8 |
| **GDS** total score (0-15 points) | 1±1/ 3.5±2.5 | 1.5±1.5/ 2.5±2 | 1.5±1.5/ 2.5±2 |
| **BDI** total score (0-63 points) | 9±0/ 14±9.5 | 1.5±2/ 8.5±8 | 3.5±4.5/ 8.5±6.5 |
| **BAI** total score (0-63 points) | 5.5±2.5/ 6±6 | 2±1.5/ 4±4 | 2.5±3.5/ 3±3 |
| **FAQ** total score (0-30 points) | 0.5±1/ 2±5 | 0±0/ 2±3.5 | 0±0.5/ 1±0.5*** (0.2 vs 0.9) (p=0.04)** |
| **Visual analogue scale question 1 memory** (0-10 points) | 4.5±1.5/ 4±1.5 | 6±2.5/ 5±2 | 5.5±2.5/ 6±2 |
| **Visual analogue scale question 2 digestion** (0-10 points) | 7±3/ 7±2.5 | 8±2/ 7±3.5 | 8±2/ 7.5±3 |
| **Visual analogue scale question 3 overall health** (0-10 points) | 7.5±1.5/ 7±2 | 8.5±1/ 7.5±2.5 | 7.5±1.5/ 8±1.5 |
| **Visual analogue scale question 4 sleep** (0-10 points) | 5.5±2.5/ 6.5±3 | 7±2.5/ 7±2.5 | 5.5±3/ 8±2.5 |
| **Visual analogue scale question 5 feeling of anxiety** (0-10 points) | 1±1/ 2±2 | 0.5±1/ 1.5±2.5 | 0.5±1/ 1.5±2 |
| **Visual analogue scale question 6 tiredness** (0-10 points) | 4±2/ 4±3 | 4±2/ 4±2.5 | 4±1.5/ 3.5±2.5 |
| **Visual analogue scale question 7 pain** (0-10 points) | 3.5±2/ 2.5±3 | 2±2.5/ 3±2.5 | 2.5±2.5/ 3.5±2 |

group PROPLA (A): probiotics first, placebo later; group PROPLA (B): placebo first, probiotics later, GDS – Geriatric depression scale, BDI – Beck depression inventory, BAI – Beck anxiety inventory, FAQ – the Functional Activities Questionnaire. Data are presented as mean ± standard deviation, p- the value shows the probability (probiotics versus placebo)

**Supplementary Table 5 Results of personal body characteristics and physical fitness and their comparisons between the online groups PROPLA and PLAPRO at several visits**

The differences of all the measures were not significant between both groups at all visits except of those in bold and with asterisks.

|  | **group PROPLA (A1) /** **group PLAPRO (B1)** | **group PROPLA after probiotics (A3)/** **group PLAPRO after placebo (B3)** | **group PROPLA after placebo (A4)/** **group PLAPRO after probiotics (B4)** |
| --- | --- | --- | --- |
| **Visit** | **Visit 1 (plus Visit 2)**  **baseline** | **Visit 3 (assessments after three months)** | **Visit 4 (assessments after three months)** |
| **Number of participants** | 6/10 | 5/5 | 6/7 |
| **Intervals from baseline assessments** (days) | 0 | 94±7/ 91±7 | 91±2/ 96±8 |
| **Weight** (kg) | 84.5±21/ 69±13.5 | 79±12.5/ 62±9*** (78.7 vs 61.8) (p=0.03)** | 84.5±20/ 64±12.5*** (84.5 vs 64.1) (p=0.047)** |
| **Body Mass Index (BMI)** | 28.5±5.5/ 24.5±4.5 | 26.5±2.5/ 24±2.5 | 28.5±5.5/ 24.5±4.5 |
| **Body water percentage** | 41.5±6/ 46.5±5.5 | 43±9.5/ 45±2 | 43.5±7/ 48.5±6.5 |
| **Muscle mass percentage** | 32±4.5/ 34±4 | 36.5±6.5/ 33.5±2 | 32±4/ 34.5±3.5 |
| **Bone weight** (kg) | 4.5±1.5/ 4.5±1 | 4.5±1/4.5±1 | 5.5±1/ 5±0.5 |
| **Number of lifting the one-kilogram dumbbell in 30 seconds** | 27.5±5.5/ 21±4*** (27.3 vs 20.9) (p=0.02)** | 27±7/ 21.5±4.5 | 26.5±5/ 23.5±4.5 |
| **The time to walk 34 meters** (seconds) | 24±2.5/ 29±9.5 | 24.5±2.5/ 29±9.5 | 23.5±2/ 25.5±2 |
| **Number of repeated standing up and sitting on a chair in 30 seconds** | 12.5±2.5/ 11±3 | 13.5±2.5/ 12±3 | 14±3/ 13±3 |

group PROPLA (A): probiotics first, placebo later; group PROPLA (B): placebo first, probiotics later. Data are presented as mean ± standard deviation, p- the values show probabilities (probiotics versus placebo)

**Supplementary Table 6 Symptoms monitored at visits and divided per probiotic and placebo groups**

| **Visit** | **Visit 1 (plus Visit 2)**  **baseline** | **Visit 3 (assessments after three months)** | **Visit 4 (assessments after three months)** |
| --- | --- | --- | --- |
|  | **group PROPLA (A1) /** **group PLAPRO (B1)** | **group PROPLA after probiotics (A3)/** **group PLAPRO after placebo (B3)** | **group PROPLA after placebo (A4)/** **group PLAPRO after probiotics (B4)** |
| **Number of participants** | 40/32 | 37/30 | 37/30 |
| Constipation | 5/3 | 0/6 (p=0.04) | 1/5 (p=0.046) |
| Diarrhea | 1/3 | 3/0 | 2/1 |
| Meteorism | 5/8 | 1/1 | 2/2 |
| Insomnia | 6/3 | 7/1 | 7/3 |
| Cramps | 1/0 | 1/0 | 1/0 |
| Thirst | 0/0 | 2/0 | 2/0 |
| Headache | 3/1 | 3/3 | 0/1 |
| Allergies | 1/3 | 3/3 | 11/3 |
| Nausea | 0/0 | 0/0 | 0/0 |
| Vomiting | 0/0 | 0/0 | 0/0 |
| Dysphagia | 0/2 | 0/0 | 0/0 |
| Infections | 0/0 | 3/1 | 2/0 |

group PROPLA (A): probiotics first, placebo later; group PROPLA (B): placebo first, probiotics later.

**Supplementary Table 7 Overview of drop-outs during the trial**

| **Last completed visit** | **Reason for withdrawal** | **Treatment group** | **In person/Online testing** |
| --- | --- | --- | --- |
| Visit 1 | Ruptured hemorrhoid | Placebo/Probiotics | In person |
| Visit 2 | Discomfort during blood sampling | Placebo/Probiotics | In person |
| Visit 2 | Gastro-esophageal reflux | Probiotics/Placebo | In person |
| Visit 2 | Constipation | Probiotics/Placebo | In person |
| Visit 2 | No longer willing to participate | Probiotics/Placebo | In person |
| Visit 2 | Constipation | Placebo/Probiotics | In person |
| Visit 3 | Operation and hospital stay | Placebo/Probiotics | Online |
